# Supplementary material for: Optimizing the microscopic agglutination test (MAT) panel for the diagnosis of Leptospirosis in a low resource, hyper-endemic setting with varied microgeographic variation in reactivity
Source: PLoS Negl Trop Dis. 2021 Jul 1;15(7):e0009565. doi: 10.1371/journal.pntd.0009565 (PMC8279374; doi:10.1371/journal.pntd.0009565)
Supplement: Attachment — Submitted filename: MAT -less resorce settings Sri lanka.docx [file pntd.0009565.s001.docx]

**Optimizing the microscopic agglutination test (MAT) panel for the diagnosis of Leptospirosis in a low resource, hyper-endemic setting with varied microgeographic variation in reactivity**

**General observations :**

The microscopic agglutination test (MAT) is the basis or cornerstone test for serological classification and diagnosis. However, in the recent past MAT is used for the sero- epidemiological studies than diagnosis. MAT antibodies appear at the end of the first week of the onset of the disease, reaches to peak during the second week or early third week. Hence detecting MAT antibodies using MAT may not much useful for the clinical management of patients . More so single MAT may not provide conclusive information on current clinical infection or confirmatory diagnosis and technically cumbersome. Molecular genetic-based techniques with a conjunction of IgM ELISA (for early and late reporting cases respectively ) are in use to provide the highest patient care. Authors themselves expressed the similar opinion. Nevertheless, MAT is the reference test and the choice for serological classification and tracking of animal vectors**.** Optimizing the panel of serovars / strains for the microscopic agglutination test (MAT) for the diagnosis of Leptospirosis (use of representative strains/ serovars reported (one or two strains from each serogroup ) and inclusion local isolates is well known and not a new . More so geographic genomics - gene acquisition and gene loss on evolutionary time scale is a continues process in leptospires and it is evidence from classification – about 300 serovars and several species (sensu stricto ). In view of the above phenomenon , optimizing MAT panel at particular point of time may not be ideal or optimized panel at another point of time.

**Specific points :**

**Introduction- Line 92 – 94:** No single MAT titre can be regarded as diagnostic of acute or current clinical infection , in a proportion of confirmed patients may have low MAT titres or past infection may	have a high titre ( it is well known that	microscopic agglutinating antibodies are long-lasof ting and persist several months to several years ). More so , prevalence of microscopic agglutinating antibodies are commonly observed among the community , use of single MAT may increase the false positive results . Since the central dogma of serology is defined as four-fold rise in titre or greater rise in titres, demonstration of rising titres by using paired MAT only confirm the current infection . More so it is observed that the dilutions are mentioned as 1: 400 or 1: 800 or so on. Please be noted here that In the diagnostic serology, the dilutions should be expressed as 1 in 400 or 1in 800 or so on as 1 in 400 is different from 1: 400 . (1 part of serum and 399 parts diluent and 1 part of serum and 400 parts diluent respectively) . Can be please rewritten for better understanding.

**Methodology : Line 183 -186 :** Please see general observations	-	MAT panel for the diagnosis of leptospirosis and please clarify .

**Results :** **Lines 235-240**: It is stated that 19 of the 150 patients with paired samples had a titre of 1 in 400 or more in the acute sample. Subsequently it is stated that 15 patients had a titre of 1 in 400 or more, but did not meet the other criteria for a positive diagnosis. I assume that 4 patients had an initial titre of 1 in 400 or more, but showed 4 fold rise in titre in the convalescent sample. If this is so, it may not be clear to the reader. Please state this clearly.

**Lines 247 – 251**: ‘We predicted the number of positives, if paired samples were available for all patients…’. Does this mean that the authors applied the proportion of positive cases among the 150 patients (33%) to the entire cohort of 982 patients to estimate the number of cases among them? If so, please clarify this and state the estimated number.

**Lines 249-251**: Did the authors calculate the 95% confidence interval of the estimated number? What is 29%? Is it the width of the confidence interval of the estimated prevalence of leptospirosis among the 150 patients whose paired sera was available? Please state the confidence limits as lower and upper bounds.

**Line 250-251**: It is not clear what ‘difference’ was statistically significant. How McNemar test, which is for paired data, was performed? . Please clarify .

**Line 253 , table 2** : A total of 107 patients confirmed as positives	and out which 73 based on single MAT . Since the study was conducted in hyperendemic area out 73 confirmed patients , there is a probability that the proportion these 73 cases may be false positives (please see Specific points mentioned – Introduction -line 92 – 94 ) . The base line MAT titres in the community may vary from one geographical area to other . Is there any community based study conducted at the study area	to estimate the base line titre and to draw cut -off titre for single MAT . Please clarify .
