# Supplementary material for: Optimizing the microscopic agglutination test (MAT) panel for the diagnosis of Leptospirosis in a low resource, hyper-endemic setting with varied microgeographic variation in reactivity
Source: PLoS Negl Trop Dis. 2021 Jul 1;15(7):e0009565. doi: 10.1371/journal.pntd.0009565 (PMC8279374; doi:10.1371/journal.pntd.0009565)
Supplement: Attachment — Submitted filename: Author_Responce.docx [file pntd.0009565.s002.docx]

**Author's Responses to Reviewer Comments**

Reviewer #2: General Observations:

**Specific points:

Introduction-  Line 92 – 94:  No single  MAT titre can be regarded as diagnostic of acute or current  clinical infection,   in a  proportion of confirmed patients may have low MAT titres  or past infection may   have a high titre (  it is well known that   microscopic  agglutinating antibodies are long-lasof ting and persist several months to several years  ).  More so , prevalence of  microscopic agglutinating antibodies are commonly observed  among  the community , use of single MAT may  increase the false positive results .  Since the central dogma of serology is defined as four-fold rise in titre or greater rise in titres,  demonstration of  rising titres by using paired MAT  only confirm the  current infection .   More so it is observed  that  the dilutions are mentioned as 1: 400 or 1:  800  or so on. Please be noted here that In the diagnostic serology,  the dilutions should be expressed as 1 in 400 or 1in 800 or so on as  1 in 400  is  different from 1: 400 . (1 part of serum and 399 parts diluent and  1 part of serum and 400 parts diluent respectively) . Can be please rewritten for better understanding.**

We agreed that to prove an acute infection, four fold rise in MAT titre is better than a single high titre. However, taking paired samples is challenging as many patients don’t turn up for the convalescent sample collection. More so LERG criteria has also defined a laboratory confirmed case of leptospirosis in MAT titre of ≥ 1in 4oo in single or paired sera together with consistent clinical signs and symptoms. Hence we took single, high MAT titre as a confirmed case of leptospirosis.

MAT titre values were rewritten as suggested by the reviewer.

**Methodology : Line 183 -186 : Please see general observations   -   MAT  panel for the diagnosis of leptospirosis  and  please  clarify .**

While the point made by the reviewer is valid and we are also in agreement that this is not the best clinically valid test. However, in Sri Lanka and many other places, single sample MAT is being used as a test for clinical diagnosis. We carried out the optimization of MAT panel for Sri Lanka as it hasn’t been attempted at any occasion in the past. As per the reviewer’s opinion, an optimized panel at one particular time may not be ideal at another time. We expect to further optimize the panel by repeating the test with our new local isolates and ongoing culture isolation studies would detect newly emerging species or strains and the panel could then be revised accordingly. This is an initiative for such an ongoing process.

**Reviewer #3: The theme was well contextualized in the introduction subsidizing the development of the manuscript. The importance this work has it’s from the optimization of MAT with the proposal to change the panel of serovars used mainly in the countries with limited resources, reduction of the number of strains tested without loss of reactivity and epidemiological assessment of the disease. The etiological agent, phenotypic and genotypic classification, diversity, occurrence and it interaction with different hosts were referenced; difficulty in early diagnosis (acute phase) of leptospirosis. The definition of cases for non-endemic areas was considered as proposed by “Leptospirosis Reference Epidemiology Group”. Also, complexity of its implementation, limitations and importance of MAT, as well as the advantages and application of the techniques available for the diagnosis of leptospirosis. 
The purpose of the study is clear. The study design was appropriate to achieve the objectives of the study. The authors will use samples from a cohort of patients with undifferentiated fever. These were divided into two types/groups: the first group of patients was with undifferentiated acute fever treated in outpatient department, hospital wards and paediatrics wards with ≥ 12 years old; the second group were patients with suspect symptoms of leptospirosis from any ward, without age restriction. The cohort study was from June 2016 to January 2019 in which the samples came from geographic areas with different characteristics (temperature, altitude, ecology, rainfall, human behavior and endemicity). The study observed the ethical requirements. 
Considering that Sri Lanka is a hyperendemic country for leptospirosis, is the cut-off point 1:100 or 1:50? Why was the cut-off point 1:100 used in the screening test while in titration it was used 1:50?**

First we thanks for the elaborative comment.

The initial dilution used for both screening and run out test was 1 in 50. This was corrected in the methodology section from line 196 to 203. The cut off point used in the study was 1 in 400 as mentioned in line 93 of methodology section. We started from 1 in 50 dilutions to look for seroconversions and four fold rise in titres.

**Results
Reviewer #2: Results :**  

**Lines 235-240: It is stated that 19 of the 150 patients with paired samples had a titre of 1 in 400 or more in the acute sample. Subsequently it is stated that 15 patients had a titre of 1 in 400 or more, but did not meet the other criteria for a positive diagnosis. I assume that 4 patients had an initial titre of 1 in 400 or more, but showed 4 fold rise in titre in the convalescent sample. If this is so, it may not be clear to the reader. Please state this clearly.**

The reviewer’s assumption is correct and out of 19 paired sera samples 15 had 1 in 400 titre in the acute stage but didn’t show a four fold rise in convalescent sample. However these samples were taken as positive considering the single high titre of more than 1 in 4oo in the acute stage. The sentence was revised and rewritten from line 245-246.

**Lines 247 – 251: ‘We predicted the number of positives, if paired samples were available for all patients…’. Does this mean that the authors applied the proportion of positive cases among the 150 patients (33%) to the entire cohort of 982 patients to estimate the number of cases among them? If so, please clarify this and state the estimated number. 
Lines 249-251: Did the authors calculate the 95% confidence interval of the estimated number? What is 29%? Is it the width of the confidence interval of the estimated prevalence of leptospirosis among the 150 patients whose paired sera was available? Please state the confidence limits as lower and upper bounds.**

**Line 250-251: It is not clear what ‘difference’ was statistically significant. How McNemar test, which is for paired data, was performed? .  Please clarify .**

We removed this section as it may not be correct to include it as a part of results.

**Line 253 , table 2  :  A total of  107 patients  confirmed  as positives   and out which 73 based on single MAT . Since the  study  was conducted in hyperendemic area  out  73  confirmed patients , there is  a  probability that  the  proportion these  73  cases  may be false positives (please see Specific points mentioned  – Introduction -line 92 – 94 ) . The base line MAT titres  in the community may vary from one geographical  area to other .  Is there any community based  study conducted at the study area   to estimate the  base line  titre  and to draw cut -off  titre for single MAT . Please clarify .**

This is a dilemma we also had and thanks for pointing it out. We agree that the baseline titre is an issue. Unfortunately, there are no data to show the baseline titre levels in Sri Lanka. Theefore we used the standard definition of Mat titre used in many studies. We have added this as a limitation in the end of the discussion section

**Reviewer #3: The characterization of patients does not refer to the two types (groups) of patients initially presented (lines 157 to 166). The clinical characteristics and the disease course are extremely important for results interpretation of MAT as well as other methodologies.
I recommend adding stratify the samples evaluated by MAT according to the disease course (days or weeks after the onset of symptoms).**

The two types of patient groups are just to show that the samples used in this study are not uniform. The two groups are not actually different from each other, because many of the patient in group 1 were also probable leptospirosis patients. To make it clear, we have expanded the methods section.

**The authors used samples with less then 15 days. MAT shows reactivity from the 7th or 8th day of onset symptom and there is a considerable difference in sensitivity before and after the first week of illness. The analysis of the results considering the first and second week of illness could provide different information. This method is the gold standard recommended by the WHO as well as by other institutions and entities such as CDC and ILS.**

The comment was absolutely correct. This analysis was done focusing the clinical applicability and assuming all patients admitting to hospital will have the first sample as “acute” sample. This was the reason for not providing the duration of fever disaggregated data. And it was also not a focus of this analysis.

**Was other methodology performed (PCR, ELISA) in parallel to MAT of the two types/groups of patients studied?**

Though the PCR was performed it was not according to the two types of patients.

**Table 1 shows data from 982 samples from 1132 patients. Of the total samples, 105 patients did not report age, 79 not report sex, 78 ethnicity, 7 patients were lost without information about they were hospitalized or were on an outpatient departments and about the clinical presentation, 227 patients not even were mentioned if they presented other symptoms.I recommend completing the information even when it has not been properly reported.**

It was from 1132 samples from 982 patients. We agree with this comment fully that there are missing data in many instances. Unfortunately, this was the reality of the database where the missing information rates were high. There was a reason for these missing data. A set of samples we received from “possible” patients included limited information. Only those who were recruited under group 1 we had most data. The reason for not doing further analysis using these characteristics was primarily due to this reason and possible issues of biases when the missing information is high. However, the objective of this study was not affected by that.

**Under the table 1, line 237, the three classic characteristics (symptoms) are referenced: headache, myalgia and fever. I recommend that, in the same way, the two classic characteristics (symptoms) be referenced (myalgia and fever?).**

It was any two out of three. We have added this information in the revised manuscript.

**The line 239 mention of  the 150 paired samples, 19 (13%) had titres of ≥1:400 during the acute stage and lines 240-241 mention another 11 (7%) samples had titres ranging from 1:50 to 1:200. Paired sample analysis showed 49 (33%) confirmed cases. I can conclude that, of the 49 paired samples 19 it were from the convalescent phase?
I recommend conceptualize the acute phase by defining the period in days and rewriting the text from lines 239 to 244 with the exception of the last sentence to improve understanding.
The writing of the line 243 is not clear “and another 15 (10%) with titres ≥1: 400 but not falling into the above two categories”.**

This section is rewritten.

**In Table 2, which type of patients according to the group mentioned in lines157 to 166 does the 107 leptospirosis confirmed patients belong to?**

Please see the clarification above

**Table 3 show that were used serovars of 17 serogroups recommended by the WHO, but there are different serovars and strains from the recommended panel. If were used the exactly recommended serovars and strains, could it generate a different reactivity profile?**

We used the CDC panel which includes 16 out of 17 serogroups recommended by WHO except the serogroup Panama. Due to problems in long term maintenance 2 serogroups; Javanica and Cynopteri were not included (mentioned in lines 190-193). This may be a short coming of the study, but we included 5 additional local strains which included a serovar in the serogroup Javanica (L. borgpetersenii serogroup Javanica serovar Ceylonica str. Piyasena). Considering the initial appearance of broad reacting antibodies in the early phase of the illness, we assume though the serovars and strains which were used in this study is slightly different from WHO panel, the interference with the sera reactivity would be minimal.

**Please review the wording for lines 239 and 290. The line 239 mention that is 19 positive paired samples while the line 290 mention 15 positive paired samples in the acute phase? Which is the correct number of sample?**

The number 15 was the number with acute sample positive and convalescent having a higher titre. This correction was done in the manuscript.

--------------------

**Conclusions**

**Reviewer #1: Despite the shortcomings of the MAT, well described here, also ELISA tests do have shortcomings (ie cross reactions). This also should be adressed in the discussion, to avoid that readers use any ELISA which tests for antileptospiral antibodies.**

A component on other serological tests was included from line 445-451

**Reviewer #2: A total of  107 patients  confirmed  as positives   and out which 73 based on single MAT . Since the  study  was conducted in hyperendemic area  out  73  confirmed patients , there is  a  probability that  the  proportion these  73  cases  may be false positives (please see Specific points mentioned  – Introduction -line 92 – 94 ) . The base line MAT titres  in the community may vary from one geographical  area to other .  Is there any community based  study conducted at the study area   to estimate the  base line  titre  and to draw cut -off  titre for single MAT . Please clarify .**

No reported community based studies has been carried out in the study area to estimate the baseline titre. However together with suggestive history, clinical signs, symptoms and single high titre of more than 1 in 400 is very likely to be due to acute leptospirosis rather than due to persistent antibodies from previous infections though we can’t exclude it completely.

**Reviewer #3: Manuscript discussion emphasizes the difficulties in performing the MAT technique. Considering that, the objective of the article was “The purpose of the present study was to describe the procedure for selecting a sensitive as well as cost effective MAT panel in Sri Lanka using a cohort of patients with acute undifferentiated fever”.
I recommend including in the discussion the limitations of the other diagnostic methods, mainly the serological tests.
In this study, were the samples submitted to analysis by another methodology? If so, it would be important to present these data.**

No other serological tests were done on these samples to make any comparison.

Discussion was modified to include limitations of other serological tests from line 445-451

--------------------

Editorial and Data Presentation Modifications”. 

**Reviewer #1: 1. When reading the procedure of the MAT, it seems that the authors did not include the volume of the leptospiral antigen in the titers. They describe the serum sample is diluted 1:50. They also describe their dilution series ranging from 1:50 to 1:3200. In the results titres of 1:50 are described. To my knowledge most of the laboratories performing MAT report titers including the added antigen. I would suggest the authors to state cleary that they report the serum dilutions, without the antigen, so that this is clear to all readers.**

The lines from 196-203 were written under the section “Sample processing and MAT procedure” to make it clear that the final dilution of serum(after addition of the antigen) used for the study was from 1 in 50 to 1 in 3200.

**2. The sentence from line 257 to 258 needs to be reformulated. It seems the authors mean that from the panel of leptospiral strains, there was with each serovar a MAT titer with 1 or more of the 982 serum samples. Or they should say that by MAT panel they mean the sera tested by MAT. But earlier in the methods (line 183) they state that the MAT panel consists of leptospiral serovars.**

Edited

**3. strain Van Tienan should be spelled as Van Tienen.**

Done. Thanks

--------------------
